# MANAGING ANXIETY DURING THE COVID-19 PANDEMIC: THE SIGNIFICANCE OF MEDITATION AND COMPASSIONATE LOVE

**DOI:** 10.1093/geroni/igad104.1269

**Published:** 2023-12-21

**Authors:** Nirmala Lekhak, Tirth Bhatta, Eva Kahana, Joel Snyder

**Affiliations:** University of Nevada, Las Vegas, Las Vegas, Nevada, United States; University of Nevada, Las Vegas, Las Vegas, Nevada, United States; Case Western Reserve University, Cleveland, Ohio, United States; University of Nevada, Las Vegas, Las Vegas, Nevada, United States

## Abstract

**Background & Significance:**

Prior research has consistently established the role of meditation as a coping resource to mitigate the adverse impacts of stressful life situations on mental health. Yet, our understanding of mechanisms that transmit the positive effects of meditation on mental health among older adults is limited. Specifically, little research has examined the impact of meditation on positive emotions such as compassionate love and its subsequent effect on later-life psychological well-being. Purpose: We rely on Fredrickson’s broaden-and-build theory of positive emotions to assess the role of compassionate love in mediating the relationship between meditation practice and mental health.

**Methods:**

Using data from our nationwide web-based survey (n=1,861), we conducted a mediation analysis to assess the indirect effect of meditation on mental health outcomes (depressive symptoms measured with a 10-item Center for Epidemiologic Studies Depression scale and anxiety measured with a 6-item PROMIS Emotional Distress-Anxiety Scale) via compassionate love.

**Results:**

Our ordinary least squares regression (OLS) model estimates suggest that older adults who practiced meditation (compared to those who did not) had significantly higher feelings of being loved (b = 0.11, p<0.05) and those who experience more love had lower symptoms of depression (b = -2.10, p<0.001) and anxiety (b = -0.99, p<0.001). We documented significant indirect effects of meditation (via compassionate love) on depressive symptoms (b = -0.23, p<0.05) and anxiety (b = -0.11, p<.05). Conclusion/Implications: Our study underscores the need for contemplative interventions that can foster compassionate love to improve mental health among older adults.

